# Conjugated linoleic acid modulation of risk factors associated with atherosclerosis

**DOI:** 10.1186/1743-7075-5-22

**Published:** 2008-08-21

**Authors:** Yukiko K Nakamura, Nichole Flintoff-Dye, Stanley T Omaye

**Affiliations:** 1 Environmental Sciences Graduate Program, University of Nevada, Reno 89557, USA; 2 Department of Nutrition, University of Nevada, Reno 89557, USA

## Abstract

Conjugated linoleic acid (CLA) has been the subject of extensive investigation regarding its possible benefits on a variety of human diseases. In some animal studies, CLA has been shown to have a beneficial effect on sclerotic lesions associated with atherosclerosis, be a possible anti-carcinogen, increase feed efficiency, and act as a lean body mass supplement. However, the results have been inconsistent, and the effects of CLA on atherogenesis appear to be dose-, isomer-, tissue-, and species-specific. Similarly, CLA trials in humans have resulted in conflicting findings. Both the human and animal study results may be attributed to contrasting doses of CLA, isomers, the coexistence of other dietary fatty acids, length of study, and inter-and/or intra-species diversities. Recent research advances have suggested the importance of CLA isomers in modulating gene expression involved in oxidative damage, fatty acid metabolism, immune/inflammatory responses, and ultimately atherosclerosis. Although the possible mechanisms of action of CLA have been suggested, they have yet to be determined.

## Conjugated linoleic acid

**Figure 1:**
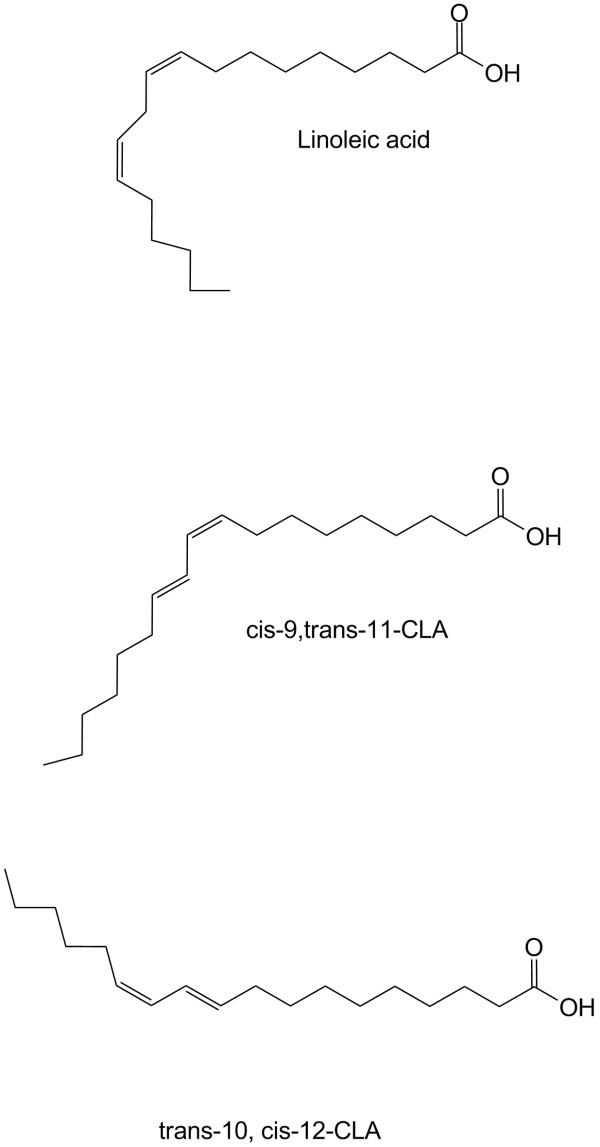
Chemical structures of linoleic acid and isomers of conjugated acid (CLA).

A group of *trans*-fatty acids, conjugated linoleic acid (CLA) has been purported to have diverse physiological functions and potential health benefits [1-6]. These unique geometric and positional isomers of octadecadienoic acid derived from linoleic acid (18:2n-6) have been found in only a limited number of foods or food products mostly derived from the fat of range animals. The highest levels of CLA are found in ruminant animals (beef, lamb and dairy cows) with beef, milk-fat, and cheese, the most common animal products containing CLA. During the biohydrogenation of linoleic acid to stearic acid, CLA is synthesized in the rumen as an intermediate by gram-negative bacteria, *Butyrivibrio fibrisolvens *[7]. CLA is also found in fish, monogastric animal products, and plant products, however, in much lower concentrations [3]. CLA isomers have been identified during the hydrogenation of fat, *e.g*., margarine production, and are found primarily in foods considered high in fat. Also, CLA is found in low concentrations in the lipids of human blood, tissue, and milk [8], presumably from dietary intakes. Although there are 28 different CLA isomers, the *cis*-9, *trans*-11 CLA isomer is predominantly found in the ruminant foods discussed earlier and accounts for >90% of CLA intake in the human diet [9]. The structures, shown in Figure 1, consist of 18 carbon atoms with two conjugated double bonds separated by a single bond, unlike linoleic acid, which is a non-conjugated diene [1]. The conjugated double bonds of CLA isomers contribute to their higher susceptibility to autioxidation than the non-conjugated bonds of linoleic acid [10]. Differences in chain length, degree of unsaturation, and position and stereoisomeric configuration of the double bonds affect fatty acid oxidation or lipid peroxidation. Usually, long-chain fatty acids are oxidized more slowly and unsaturated fatty acids are oxidized more rapidly than are saturated fatty acids. Lauric acid is highly oxidized, but PUFAs and monounsaturated fatty acids are fairly well oxidized [11]. Oxidation of the long-chain, saturated fatty acids decreases with increasing carbon number.

Many research groups have looked at the possibility of CLA isomers as anti-carcinogens. Most anti-carcinogens are plant products (phytochemical), therefore, CLA isomers are unusual find because it occurs in the highest concentration in animal products (zoochemical) with only trace amounts found in plant lipids. The possibility of CLA isomers working as a feed efficiency supplement and a lean body mass supplement has also been examined, along with its role in cancer prevention and stimulation of the immune system.

With regard to potential health benefits, considerable attention has been given to anti-carcinogenic effects of CLA isomers; however, less attentions has been devoted toward its usefulness in preventing and reversing atherosclerosis and related diseases. The majority of research studies have been done using experimental animals and *in vitro*, with only recent investigations showing the effects of CLA isomers on humans. The purpose of this review is to assess and summarize current literature and knowledge on the possible health benefits of CLA isomers, particularly with respect to atherosclerosis as a chronic inflammatory disease.

## Pathology/etiology of atherosclerosis

Cardiovascular disease (CVD) is a major cause of death in developed countries, and most cardiovascular events are secondary to atherosclerosis [12]. CVD causes high medical costs and losses of productivity. The high incidence of CVD mortality and morbidity and the economic toll of CVD emphasize the need for prevention and management of CVD associated risk factors. Although the risks for CVD are multifactorial, the three most important modifiable risk factors for atherosclerosis are: 1) smoking, 2) inactive lifestyle, and 3) elevated blood cholesterol levels from dietary sources. Of particular concern for the elevated blood cholesterol is increased low-density lipoprotein (LDL). Results of extensive epidemiological and clinical research support the direct association between elevated blood cholesterol and CVD risk.

**Figure 2:**
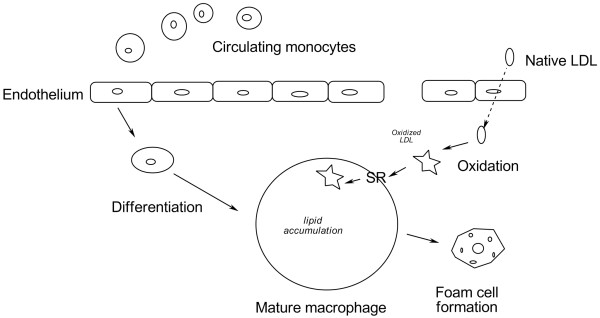
**Vascular events leading to the development of atherosclerotic lesions. **Low density lipoprotein (LDL), scavenger receptor (SR).

Atherosclerosis is a condition characterized by degeneration and hardening of the walls of the arteries and sometimes the valves of the heart. There is accumulation of lipids and other materials in the arteries which contributes to hypertension and vice versa. Figure 2 schematically illustrates the major points of oxidized LDL in the process of plaque formation. The process of atherosclerosis begins with buildup of soft fatty streaks along the inner arterial walls often at branch points. With age, fatty streaks steadily grow and become hardened with minerals, leading to plaque. Consequently, plaque stiffens and narrows the artery lumen. By middle age, most people have well-recognized plaque formation [13]. Blood platelets respond to plaque as if it was a blood vessel injury produce clots which unlike the normal blood clotting events, do not readily dissolve and instead stick to the plaque, grow and restrict blood flow, *i.e*., thrombosis. Platelet activity is under the control of eicosanoids synthesized from 20-carbon omega-6 and omega-3 fatty acids, such as prostaglandins and thromboxanes. Complication may occur when blood clots break free from the walls of arteries and make their way to a smaller artery, and shut off the blood supply to tissue; this produces an embolism.

LDL oxidation is thought to be the first step of atherogenesis, followed by foam cell, fatty streak, and plaque formation. It has been hypothesized that LDL can be transported through endothelial tight junctions and/or endothelial transcytosis from the lumen into the intima [14], in which blood antioxidants are unlikely to be available, and undergo atherogenic oxidative changes. Modified LDL is then taken up by macrophages through multiple pathways. Minimally oxidized LDL (MM-LDL) is recognized by CD14 and toll-like receptor-4 (TLR4) [15], while oxidized LDL binds to scavenger receptors (*e.g*., CD36, CD68, SR-A1, SR-B1). Aggregated forms of either MM-LDL or native LDL are endocytosed by activated macrophages [16].

Both oxidized LDL and activated macrophages by oxidized LDL uptake affect gene expression in neighboring endothelial cells (ECs), contributing to further atherogenic/inflammatory processes. Studies have documented that the oxidized LDL affect the pattern of gene expression in ECs, leading to up-regulated expression of target molecules. The oxidized LDL-induced molecules in ECs include monocyte chemoattractant proteins (MCPs), macrophage colony stimulating factors (M-CSFs), and cell adhesion molecules (CAMs) [17,18]. MCPs and M-CSFs are induced by MM-LDL, and are released from ECs. MCPs recruit monocytes to the ECs. M-CSFs promote the differentiation and proliferation of monocytes to macrophages (Figure 2). CAMs, cell surface proteins, are involved in binding with other cells or the extracellular matrix. These molecules contribute to foam cell formation by the recruitment of circulatory monocytes into vascular walls and by the stimulation of monocyte differentiation to macrophages.

The differences in genetic susceptibility to atherosclerosis were investigated using animal and human models [19-22]. ECs from the atherosclerotic prone strain of C57BL/6J mice exhibited dramatic induction of MCP-1 and M-CSF in response to MM-LDL, while ECs from the atherosclerotic resistant strain of C3H/HeJ mice showed little or no induction of MCP-1 or M-CSF. Shi *et al*. [19] provide the evidence that genetic factors influencing the endothelial response to oxidized LDL contribute to the genetic component in atherosclerosis. Levula *et al*.'s [20] microarray study reveals the groups of target genes whose expressions are altered by oxidized LDL in human macrophages. The target genes are involved in 1) lipid metabolism, 2) inflammation, growth, and hemostasis, 3) metalloproteinases and tissue inhibitors of matrix metalloproteinases, 4) enzymes, 5) structural and binding proteins, and 6) annexins. The genes involved in inflammation include M-CSF1, MCP1, and ICAM1, all of which are induced in the macrophages by oxidized LDL, and correspond to the results of previous Shi *et al*.'s EC studies.

Induced expression of CD68 and SR in human macrophages by oxidized LDL was also observed in Levula *et al*.'s microarray study. In addition, activated macrophages secrete inflammatory cytokines, such as TNF-α, that contribute to the induction of the expression of MCP-1, M-CSF, ICAM1, and VCAM1 in human aortic endothelial cells (HAECs) [21] and the expression of ICAM1 and VCAM1 in human neonatal dermal lymphatic endothelial cells (HNDLECs) [22] and the development of atherosclerosis. Thus, the pro-inflammatory gene expression in ECs is mediated by either oxidized LDL or pro-inflammatory cytokines released from activated macrophages, resulting in augmented atherogenic/inflammatory events by recruiting circulatory monocytes.

Moreover, oxidized LDL may modulate the apoptosis of vascular cells. Reeve *et al*'s [23] microarray study demonstrated that 221 genes were differentially regulated by oxidized LDL in coronary artery smooth muscle cells (CASMC). Of particular interest are apoptotic genes, FasL, Bax, and p53, induced by oxidized LDL in CASMC. Oxidized LDL induces apoptosis of ECs via the mitogen-activated protein kinase (MAPK) pathway [24]. Studies using EC and smooth muscle cell cultures demonstrated that multiple apoptotic signaling pathways were affected by ROS [25].

## Roles of ROS in atherogenesis

Reactive oxygen species (ROS) are implicated in atherogenesis. Risk factors for atherosclerosis are associated with an increased arterial wall flux of ROS that not only may oxidize biomolecules, but also directly produce phenotypic changes in vascular cells, including the induction of adhesion molecules and smooth muscle proliferation [26].

### Sources of ROS

Sources of ROS involved in atherogenesis include NADPH oxidases, nitric oxide synthases (NOS), lipoxygenases (LO), cyclooxygenases (COX), and the mitochondrial respiratory chain [27]. Native LDL is modified by ROS generated by these enzymes in vascular tissues. NADPH oxidase is composed of a number of different subunits. There are seven homologues of the gp91phox (Nox-2) subunit. Nox-4 is predominantly expressed in ECs, though the expressions of Nox-1 and Nox-2 are also detected [28]. During the respiratory burst in phagocytes, NADPH oxidase converts oxygen molecules to superoxide, which is a microbicidal oxygen metabolite. Then, superoxide is converted by superoxide dismutase (SOD) to hydrogen peroxide, which also kills microorganisms. ROS are also produced in ECs by endothelial NADPH oxidase [26,27]. Phagocytic NADPH oxidase and endothelial NADPH oxidase is one of the major ROS sources in the vasculature [27]. Monocyte differentiation to macrophage is associated with the production and the release of ROS possibly through the induction of NADPH oxidase, resulting in further LDL oxidation [29,30]. NADPH oxidase generates superoxide on the extracellular side of the plasma membrane, and the enzyme can trigger intracellular signaling by superoxide transport via chloride channel-3 [31]. Stepp *et al*. [32] reported that native LDL and MM-LDL differentially increase vascular endothelial superoxide generation in canine carotid arteries, leading to vascular dysfunction and atherogenesis. Native LDL increases superoxide by an endothelial nitric oxide synthase (eNOS)-dependent mechanism whereas MM-LDL induces greater superoxide by the mechanisms dependent on eNOS, xanthine oxidase, and NADPH oxidase. Superoxide production by vascular tissues and its interaction with nitric oxide (NO) play important roles in vascular pathophysiology. Superoxide reacts rapidly with NO, reducing NO bioavailability and producing the oxidative peroxynitrite radical [33]. Endothelial activation via LOX-1 produces additional ROS, generating a positive feedback loop for further LDL oxidation [34]. ROS generated in a NADPH oxidase-dependent pathway mediate TNF-α-induced MCP-1 expression in ECs, and the induction of MCP-1 expression is suppressed by the antioxidant enzymes, SOD and catalase [35]. Since phagocytic NADPH oxidase is the first line of the host defense system, selective suppression of vascular NADPH oxidase in local inflammatory lesions might be one of the therapeutic strategies [36].

**Figure 3:**
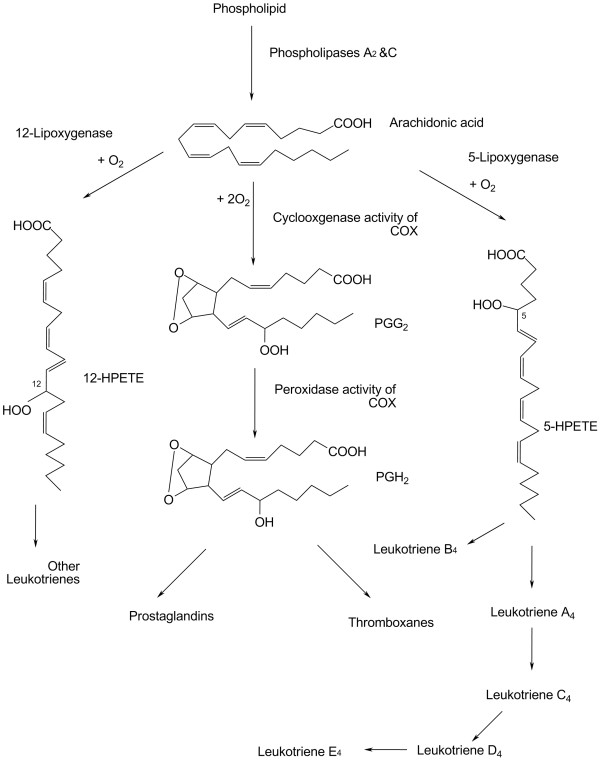
**Metabolic pathways for the formation of prostaglandins, thromboxanes, and leukotrienes. **Prostaglandin H_2 _(COX), lipoxygenase (LOX), 12-hydroperoxyeicosatetraenoate (12-HPETE), 5-hydroperoxyeicosatetraenoate (5-HPETE).

Cyclooxygenases (COX-1 or COX-2) and lipoxygenases (5-, 12-, or 15-LO) also contribute to ROS generation during arachidonic acid (AA) metabolism shown in Figure 3[27,37]. The initial products in AA metabolism are highly reactive peroxides. Overexpression or induction of COX-2 increases ROS in certain cell types [38,39] and the effects of overexpression of COX-2 are cell-/tissue-specific [40]. Constitutive COX-1 and inducible COX-2 catalyze the conversion of free PUFAs to prostanoids (prostaglandins and thromboxanes), while LO generates the leukotrienes. Prostanoids and leukotrienes comprise a large and complex family of biologically active lipids derived from PUFAs by insertion of molecular oxygen. Collectively, these compounds are termed eicosanoids. Both prostanoids and leukotrienes play important roles in inflammation. Non-esterified PUFA released from the sn-2 position (middle carbon of glycerol) of the membrane phospholipids by the action of specific phospholipases are substrates for COX, LO, or cytochrome P450 monooxygenases (CYP) [41,42]. The metabolism of AA has two main pathways: the cyclic pathway leading to prostanoid formation and the linear pathway resulting in leukotriene formation. Two molecules of oxygen are added in the first step for the generation of prostaglandin G (PGG). The second step, via the peroxidase activity of COX, converts PGG2 into PGH2, the precursor for either thromboxanes or other prostaglandins. The peroxidase step is inhibited by aspirin or ibuprofen. LO is also a major source of extracellular superoxide release in a certain cell type during AA metabolism [37]. The LO pathway is responsible for the formation of leukotrienes and hydroxyeicosatetraenoic acids (HETEs). LO isoforms act upon arachidonic acid to form 5-, 8-,12-, or 15-hydroperoxy eicosatetraenoic acids (HPETEs), which are unstable and can be reduced to the hydroxyl derivatives (HETE) *in vivo*. The range of HPETEs with biological activity is known as the leukotrienes. Both COX and LO products diffuse from cells and act locally at nanomolar levels on cell surface receptors linked to G-proteins. Activation of G-protein-associated receptors leads to changes in intracellular cAMP or calcium, which serve as second messengers that activate signaling mechanisms influencing various cellular functions. The COX products are modulators of thromboregulatory and chemotaxic responses, and inflammation. The LO products are involved in vascular permeability, vasoconstriction, and bronchoconstriction. The third route for eicosanoid production is via the CYP, in particular CYP4 family [43], including epoxy derivatives of 20:4 ω-6 that can modulate calcium signaling, channel activity, transporter function, and mitosis. This mechanism seems to be more consequential in cells when COX and LO activities are minimal.

This body of research has verified that multiple enzymes are involved in ROS generation that leads to atherogenesis. Hence, it is speculated that suppressing ROS generation may be a therapeutic target for preventing and alleviating atherosclerosis.

### Oxidation of biomolecules

ROS play a role in LDL oxidation. LDLs are rich in polyunsaturated fatty acids (PUFAs), which are susceptible to oxidation [12]. Lipid peroxidation can commence by ROS and other mechanisms that result in abstraction of an electron from a PUFA [44]. The sequential carbon-centered radical undergoes rearrangement, and in the presence of oxygen, will add oxygen to form a peroxyl radical (ROO•). Propagation of the free radical reaction can occur by reaction of the peroxyl radical with another PUFA, generating the corresponding fatty acid hydroperoxide (ROOH) and another carbon-centered radical. Other factors, such as Fe^+2^, and other oxidants can result in an amplification of the free radical process. Vitamin E is a nonenzymatic chain-breaking scavenger of lipid radicals generated in cell membranes; it protects against further lipid peroxidation. Vitamin C is an important antioxidant against lipid peroxidation because it has a high reactivity with the oxygen-centered radical. Oxygen-centered radicals have sufficient polarity to be accessible to the aqueous soluble vitamin C. Also, when vitamin E reacts with a radical, vitamin E is converted to its radical form which can be recycled to reduced vitamin E by reacting with vitamin C. Other reducing compounds such as glutathione and NADPH act in concert with vitamins E and C in an antioxidant cascade.

LDL oxidation also results in changes in apolipoprotein B epitope. The oxidized apolipoprotein portion of LDL is subsequently recognized and internalized by SR-A, whereas the oxidized lipid moiety of LDL is bound to CD36 on macrophages [45].

## Inflammation and fatty acids/other protective compounds

### Fatty acids

The relationship between fatty acids and atherosclerosis and other inflammatory diseases has been suggested by epidemiological, clinical, and in-/ex-vivo studies. Increased intake of saturated fatty acids is positively associated with development of atherosclerosis and inflammation. In contrast, omega-3 (ω-3) fatty acids, such as eicosapentaenoic acid (EPA, C20:5) and docosahexaenoic acid (DHA, C22:6), have shown protective effects against CVD. EPA and DHA are major components of dietary fish and fish oils. Like EPA and DHA, CLA isomers, exhibit protective effects against atherogenesis [46,47] and inflammatory bowel disease (IBD) [5,48,49] and antioxidant effects [50] in *in-/ex-vivo *studies; however, clinical studies have been inconclusive.

Most of the fatty acids synthesized or ingested have one of two fates: incorporation into triglycerides for the storage of metabolic energy or incorporation into the phospholipid components of membranes. The selection between the alternative fates depends on the need (*i.e*. growth and starvation) [42]. Fatty acids may differentially affect inflammatory processes and ultimately the etiology of atherosclerosis in three ways as:

1) the components of membrane; fatty acids may serve as precursors of pro- or anti- inflammatory eicosanoids;

2) the components of LDL; fatty acids may differentially modulate recognition of macrophage receptors and subsequent inflammatory processes and atherogenesis; and

3) the regulators of gene expression; fatty acids may differentially regulate inflammatory gene expression, serving as ligands for transcription factors (*e.g*. PPARs).

Almost all mammalian cells, except red blood cells, produce eicosanoids, which play a role in inflammation. Arachidonic acid (AA, C22:4) is the most important precursor of eicosanoid, and AA is synthesized from linoleic acid (LA, C18:2, ω-6) by enlongation and desaturation. In response to hormonal or other stimuli, phospholipase A2, present in most types of mammalian cells, attacks membrane phospholipids, releasing AA from the middle carbon of glycerol. Enzymes of the smooth endoplasmic reticulum then convert AA into eicosanoids, potent biological signaling molecules [42].

LA (18:2, ω-6) and α-linolenic acid (ALA, 18:3, ω-3) serve as the precursors for longer-chain ω-6 (*e.g*., arachidonic acid: AA) and ω-3 fatty acids (*e.g*., EPA, DHA), respectively. Neither ω-3 nor ω-6 fatty acids can be synthesized in mammals due to the lack of certain types of desaturases. ω-3 Fatty acids cannot be generated from ω-6 fatty acids in mammals. Hence, the source of these PUFAs is limited to dietary intake [51]. Dietary EPA, DHA, and CLA can partially replace AA derived from LA in the cell membrane [52,53]. Usually the plentiful LA may exclude these fatty acids from incorporation into membrane phospholipids [54] and/or LDL. However, EPA, DHA and CLA may influence eicosanoid production from AA and subsequent immune and inflammatory processes. For example EPA and DHA decrease the synthesis of pro-inflammatory eicosanoids, such as leukotriene-4 and prostaglandin-2 by replacing AA in phospholipid bilayers and by inhibiting cycloxygenase activity [55-57]. As components of LDL and/or membrane, these fatty acids may affect the inflammatory gene expression by altering signaling pathways. The unusual conformation structures (kinks) in unsaturated fatty acids interfere with the membrane motion [42] and possibly signal transduction. In addition, EPA and DHA reduce the expression of adhesion molecules induced by oxidized LDL in endothelial cells [58-60]. ω-3 PUFAs suppress inflammatory gene expression by inhibiting TLR4 signaling pathway, whereas saturated fatty acids exhibit the opposite effect [61], Thus, subsequently decreasing risk for CVDs.

### Phytochemicals/dietary antioxidants

Several epidemiological studies have reported an inverse relationship between intake of vegetables and fruits (in particular those rich in antioxidant vitamins including vitamins C and E and β-carotene), and risk for CVD [62-65]. The protective effects of these antioxidant vitamins on atherosclerosis have been intensively investigated in animal and human studies. According to the oxidative modification hypothesis, oxidized LDL is immunogenic and atherogenic and LDL oxidation triggers atherosclerotic processes. Therefore, the protection of LDL from oxidation may be crucial to the prevention of atherosclerosis; the antioxidant components of LDL may prevent LDL oxidation.

Vitamin E is the generic term for all tocopherol and tocotrienol derivatives that exhibit the biological activity of α-tocopherol. There are eight naturally occurring isoforms synthesized in plants. α-Tocopherol is the most biologically and chemically active form of vitamin E. The hydroxyl groups at the C-6 position of tocopherols enable them to scavenge free radicals and superoxide. Although γ-tocopherol is predominant in the American diet, its plasma levels are only 10% of plasma α-tocopehrol levels (about 25 μmol/L α-tocopherol) [66].

α-Tocopherol is the major antioxidant in LDL and one LDL particle contains approximately six molecules of α-tocopherol. α-Tocopherol in LDL plays a role in preventing LDL oxidation. Vitamin E depletion in LDL may trigger LDL oxidation; and the addition of micromolar concentrations of vitamin E inhibits LDL oxidation. All other antioxidants, such as γ-tocopherol, carotinoids, and ubiquinol-10, are present in much smaller amounts than α-tocopherol. In contrast to α-tocopherol, carotenoids play only a minor or no role in LDL protection [67]. However, many clinical studies have failed to demonstrate the protective effects of vitamin E. One explanation may be that vitamin E exhibits prooxidant activity in the absence of co-antioxidant compounds capable of reducing the tocopherol radical [68,69]. A similar situation may occur with other antioxidants, such as β-carotene [70-72]. Depending on the concentrations, environmental conditions and presence of oxygen or other oxidants, compounds with antioxidant properties may exhibit prooxidant or other non-antioxidant properties.

Polyphenolic compounds, such as resveratrol and catechins, are derived from plants, and the compounds have shown anti-atherogenic and anti-inflammatory effects. The beneficial effects of the compounds are attributed to their abilities to function as antioxidants by: 1) inhibition of prooxidant enzymes, such as lipoxygenases, cyclooxygenases, and xanthine oxidase, possibly through suppressing the activation of redox-sensitive transcription factors, NF-κB and activator protein-1 (AP-1), and 2) induction of antioxidant enzymes such as glutathione S-transferase, glutathione peroxidase (Gpx), superoxide dismutase (SOD), and catalase [73,74].

### Endogenous/enzymatic antioxidants

Antioxidant enzymes are involved in the maintenance of intracellular and extracellular reducing reactions [75] and suppress the generation of free radicals as the first line of antioxidant defense [12]. Antioxidant enzymes include superoxide dismutase (SOD) and catalase. SOD is expressed in most cell types, and converts harmful superoxide to less harmful hydrogen peroxide and oxygen. Catalase catalyzes the dismutation of hydrogen peroxide to oxygen and water. Catalase has an iron redox center. Catalase is located predominantly within peroxisomes to protect from hydrogen peroxide generated during fatty acid β-oxidation within the cellular organelles [76].

The antioxidant enzymes play a role in preventing atherogenesis. Increased expression of GR in macrophages reduces atherosclerotic lesion formation in LDL receptor-deficient mice [77]. Over-expression and/or induction of CuZn-SOD and catalase can be beneficial because of: 1) decreases in superoxide levels in ECs; 2) suppression of oxidative stress, e.g., age related; 3) protection against inflammatory events by inhibiting NF-κB activation; and 4) suppression of low-density lipoprotein (LDL) oxidation by ECs [50,76,78-81].

## Target genes/signaling pathway

Atherosclerosis is a chronic inflammatory disease with an underlying abnormality in redox-mediated signals in the vasculature [82]. ROS play a role in the signaling involved in atherogenic/inflammatory processes. There are two major redox-sensitive signaling pathways related to the atherogenic/inflammatory processes: NF-κB-, and peroxisome proliferator-activated receptor (PPAR)-mediated pathways. Fatty acids may act as gene regulators. CLA isomers are ligands with high to moderate affinity and activators of PPAR α and γ. CLA isomers may induce responsive genes of both PPAR α and γ *in vivo *[6]. The *trans*-10, *cis*-12 CLA isomer inhibits the NF-κB p50 and p65 subunits binding to DNA [83]. Also, CLA isomers may involve the control of redox status by regulating genes, whose products influence ROS generation, through transcription factors (PPARγ and NF-κB), which are concentration-dependent [84].

### NF-κB

NF-κB is a redox-sensitive transcription factor expressed in all cell types; it recognizes and binds to specific DNA sequences (5'-GGGRNNYYCC-3'). NF-κB activation is triggered by the IκB kinase (IKK)-mediated degradation of inhibitor κB (IκB), which regulates NF-κB. NF-κB is activated by intra-/extra-cellular ROS and/or ROS-modified target biomolecules, and is involved in regulating immune and inflammatory responses. NF-κB-mediated target genes include: inflammatory cytokines (*e.g*., TNF-a, IL-1, IL-2, M-CSF), chemokines (*e.g*., MCP-1), adhesion molecules (*e.g*., ICAM-1, VCAM-1), inflammatory enzymes (*e.g*., iNOS, COX-2), and apoptotic regulators (*e.g*., Fas ligand, Fas, p53) [85].

Oxidized LDL may affect atherogenesis in part via the NF-κB activation pathway. Oxidized LDL activates NF-κB as well as *C. pneumoniae *[86]. Resveratrol, an antioxidant polyphenol derived from plants, attenuates TNF-α-induced inflammatory gene expression and monocyte adhesion to human coronary arterial endothelial cells (HCAECs) by inhibiting NF-κB activation, suggesting that the anti-inflammatory actions of resveratrol are responsible for anti-atherogenic effects [78]. Oxidized LDL exerts biphasic effects on NF-κB: 1) inflammatory effects by up-regulating inflammatory gene expression via NF-κB activation at lower concentrations of oxidized LDL; and 2) immunosuppressive effects by inhibiting NF-κB activation triggered by inflammatory agents such as lipopolysaccharide (LPS) at higher concentrations of oxidized LDL [85]. HUVECs incubated with LPS which causes inflammatory gene expression via TLR4 activation, induce the expressions of TLR4, LOX-1, ICAM-I, and E-selectin, and increase monocyte adhesion to endothelium and NF-κB activation levels, suggesting the atherogenic process is mediated through TLR4/NF-kB pathways [87]. There are two types of TLR4/NF-κB pathways identified: MyD88-dependent and independent pathways. MyD88 is a common downstream adaptor molecule for most TLRs, and recruits other molecules required to activate NF-κB. Saturated fatty acids trigger TLR4 and downstream NF-κB activations, resulting in inflammatory gene expression (*i.e*. COX2 or iNOS). In contrast, unsaturated fatty acids inhibit TLR4/NF-κB activation. This inhibition may be due to the alteration of fatty acid components in membrane lipid rafts, which may lead to the disruption of the recruitment of the downstream signaling components [61].

### PPARs

**Figure 4:**
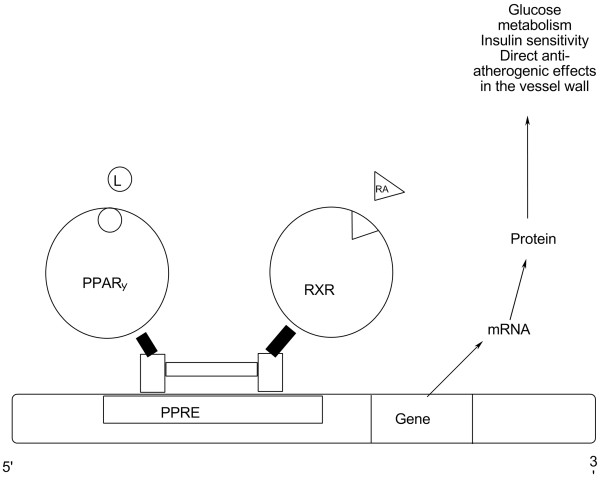
**Role of PPARγ.** Peroxisome proliferator response element (PPRE), retinoic X receptor (RXR), retinoic acid (RA), ligands (L).

PPARs (PPARα, PPARβ, and PPARγ), a group of nuclear receptors, belong to the steroid hormone receptor superfamily [88]. PPARs heterodimerize with the 9-cis retinoic acid receptor (RXR) and bind to peroxisome proliferator response elements (PPREs: 5'-AGGTCAnAGGTCA-3') which are located in enhancer sites of target genes (Figure 4). PPARα and PPARγ are expressed in vascular endothelial cells and smooth muscle cells as well as adipose tissues [79,89]. PPARα and PPARγ play a role in inflammation, adipogenesis, and insulin sensitization. Thiazolidinediones (TZDs), a group of synthetic PPARγ ligands, have shown beneficial effects as atheroprotective drugs. 15-Deoxy-Δ^12,14^-prostaglandin J2 (15d-PGJ2), an prostanoid, is a natural ligand and an activator for PPARγ. 15-LOX products, 9- and 13-hydroxy-octadecadienoic acids (HODEs), are also PPARγ activators [89]. Therefore, these eicosanoids, 15d-PGJ2 and HODEs, support the implication of PPARγ in inflammation.

PPARγ activation may be involved in oxidized LDL-induced inflammatory gene expression and macrophage lipid metabolism [45,90,91]. Although CD36 is up-regulated by oxidized LDL via PPARγ activation, PPARγ activation suppresses oxidized LDL-induced inflammatory effects by inhibiting inflammatory gene expression. Kunsch and Medforld [82] suggest that PPARγ participates in a positive feedback loop and that alternative or downstream pathways may trigger PPARγ activation resulting in anti-inflammatory effects. For example, 15d-PGJ2 is known to be an endogenous PPARγ activator. 15d-PGJ2 may be a possible anti-inflammatory mediator, though the physiological levels of 15d-PGJ2 may be insufficient to modulate PPARγ activation [92]. PPAR activators are negative regulators of macrophage activation and antagonize the activities of the transcription factors, AP-1, STAT, and NF-κB, involved in inflammatory gene expression [93,94].

Lipoprotein lipase (LPL), a lipolytic enzyme, may play an important role in regulating early atherogenesis. LPL neither acts on nor binds to oxidized LDL. LDL(-) is a form of native LDL containing intermediately modified subfractions with higher electronegative charge and is taken up by LDL receptors. Lipid peroxidation is greater in LDL(-) than in native LDL; LDL(-) exhibits inflammatory effects. Ziouzenkova *et al*. [95] demonstrated that LPL-treated LDL(-) reduced inflammatory gene expression in human ECs by suppressing NF-κB and AP-1 activations and by increasing the expression of IκB, a target gene for PPARα, via PPARα activation. In contrast, LDL(-) alone increased the inflammatory responses. 9- and 13-HODEs, both known as PPARγ activators, are released during the hydrolysis of both native LDL and LDL(-), resulting in PPARα activation and anti-inflammatory effects.

Expression of antioxidant enzymes, Cu/Zn SOD and catalase, may be modulated through both PPARγ and NF-κB activations. Possible multiple binding sites for PPARγ and NF-κB have been identified: 1) within the promoter region of *SOD1*, and 2) one binding site for NF-κB within the promoter region of catalase. NF-κB activation is associated with the induction of proinflammatory gene expression. Although Cu/Zn SOD is an antioxidant enzyme, it is induced to convert superoxide to hydrogen peroxide that is still microbicidal; it also serves as a host defense with NADPH oxidase in phagocytes. Furthermore, the treatments of possible PPARγ activators increase both PPARγ and NF-κB DNA binding activities, indicating that these two redox-sensitive transcription factors coordinate and propagate feedback loops between each other.

## Interactions between genes and diet: Risk factors

Single-nucleotide polymorphisms (SNPs) are a genetic variation of differences in a single nucleotide between individuals. The gene-diet interaction between common SNPs located in candidate genes and dietary factors related to lipid metabolism has been recently reported [96]. These candidate genes include: *APOA1 *(75G→A) encoding apolipoprotein A-I, an apolipoprotein of HDL, and *PPARA *(Leu162Val) encoding PPARα. Ordovas *et al*. and Tai *et al*. suggest specific interactions between these polymorphisms and lipid profiles. HDL-cholesterol concentrations increased significantly with increasing PUFA intake in women with the A allele (G/A and A/A) in *APOA1*, while HDL-cholesterol concentrations decreased as PUFA intake increased in women with the homologous G allele (G/G) in *APOA1 *[97]. The Leu162Val polymorphism in *PPARA *is associated with increased plasma concentrations of total cholesterol, LDL cholesterol, and apolipoprotein B [98]. Thus, these interactions influence CVD risk in different directions through effects on two different CVD risk factors: HDL cholesterol through the polymorphism in *APOA1*and triacylglycerol through the polymorphism in *PPARA*. The effects of Pro12Ala polymorphism in *PPARG2 *on type 2 diabetes and obesity are also reported. The 12Ala allele (Ala/Ala) in *PPARG2 *confers a reduced risk for type 2 diabetes and decreased obesity-associated insulin resistance in the French Caucasian population [99]. Furthermore, the associations of polymorphisms in genes involved in antioxidant defense systems, with CVD and other diseases, have been proposed. A human sodium-dependent vitamin C transporter, SVCT1, is encoded by *SLC23A1*, and mediates intestinal absorption and renal absorption of L-ascorbic acid [100]. *SLC23A1 *appears to have population-specific variants, and populations with discrete genetic variants might require different recommended values of vitamin C intake to maintain health and/or to prevent disease [101]. An antioxidant enzyme, SOD2 (Mn-SOD), is constitutively expressed in most cells. The SOD2 polymorphism, 16Val homozygous, may be a predisposing factor for lung cancer, cardiomyopathy, diabetic complications, hypertension, and CVD [102-105] and may influence longevity [106]. *GPX1 *encodes Gpx1, and may be a target gene for exploring roles of its variants in the etiology of various human diseases [107]. Genetic variations may also affect inflammatory responses. TLR4 is a pattern recognition innate immunity receptor that binds LPS found in gram-negative bacterial walls and possibly oxidized LDL [15]. The Asp299Gly TLR4 polymorphism may decrease the risk of atherosclerosis by reducing TLR4 receptor signaling and subsequent inflammatory response [61,108].

Thus, genetic variations are widely distributed in various components involved in atherogenesis. The total genetic variations between individuals may differently influence the risk for and the etiology of atherosclerosis and CVD. It may be possible to provide individuals with dietary/therapeutic guidance tailored to their genotypes, given adequate information on the interaction between specific genetic polymorphisms and diet [109]. In other words, genetic variations might predict the significant differences in disease etiology between different species, thus suggesting the limitation of animal studies. Hence, nutrigenetics and nutrigenomics would be new powerful tools for investigating the relations between diseases and genes at individual/intra-species levels.

## Experimental studies of CLA isomers

### Animal studies

#### Rabbits

The possibility that the anti-atherogenic properties of CLA isomers may influence atherosclerotic lesions and blood lipid levels has been tested in animal models.

Rabbit studies suggest protective and/or therapeutic effects of CLA isomer treatments. Rabbits fed an atherogenic diet and supplemented with CLA isomer mixture (*cis*-9, *trans*-11 CLA isomer: *trans*-10, *cis*-12 CLA isomer = 1:1; 0.5 g CLA diet/day/rabbit) had significantly less aortic fatty lesions and lower levels of plasma triglycerides and LDL-cholesterol, compared to control animals [110]. A rabbit study by Kritchevsky *et al*. [46] also reported reduced atheromatous lesions to the same extent in all CLA-fed groups (90 days): 1% (final dietary concentration) each of the *cis*-9, *trans*-11 CLA isomer, the *trans*-10, *cis*-12 CLA isomer, and the two isomer mixture, compared to control group.

A dose-dependent effect of CLA isomer mixture (*cis*-9, *trans*-11 CLA isomer: *trans*-10, *cis*-12 CLA isome r = 1:1) on atherosclerotic regression was demonstrated in two rabbit studies [47,111]. New Zealand white rabbits fed a 0.1% (final dietary concentration) CLA isomer mixture diet after receiving an atherogenic diet, showed an inhibition of atherogenesis, while rabbits fed a 1% CLA mixture diet exhibited a 30% regression of established atherosclerosis [47]. Dose-dependent regression of established atherosclerosis was seen in rabbits fed CLA isomer mixtures ranging between 1 and 10 g/kg body weight. However, both serum cholesterol and triglyceride levels were higher in CLA fed groups than in control group, despite a dose-dependent reduction of lipid levels within the range of CLA isomer mixtures [111].

#### Mice

Mouse model studies suggest the anti-atherogenic effects of CLA isomers, and some of those studies indicate that the effects of CLA are tissue- (*i.e*, hepatoxicity described later in this section), isomer-, and dose-specific.

Atherosclerotic prone strain C57BL/6 mice fed an atherogenic diet containing 2.5 or 5 g/kg body weight CLA isomer mixture (*cis*-9, *trans*-11 CLA isomer: *trans*-10, *cis*-12 CLA isomer = 1:1) for 15 weeks developed higher serum HDL-cholesterol (total cholesterol ratio and lower serum triacylglycerol concentration) than controls. However, despite causing a serum lipoprotein profile considered to be less atherogenic, addition of CLA isomer mixture to the atherogenic diet increased the development of aortic fatty streaks. Mice consuming a diet of 2.5 g CLA isomer mixture/kg body weight, but not 5.0 g CLA isomer mixture/kg body weight, developed a significantly greater area of fatty streaks than the controls [112], suggesting dose-specificity. A study by Arbones-Mainar *et al*. [113] showed isomer-specific effects on the development of atherosclerosis. The *trans*-10, *cis*-12 CLA isomer diet (1% final dietary concentration for 12 weeks fed to apolipoprotein E knockout mice) increased the values of blood lipid, an inflammatory marker (8-iso prostaglandin E), atherosclerotic plaque, and macrophage content and activation. However, the *cis*-9, *trans*-11 CLA isomer diet (1%) inhibited atherogenic development. Moreover, de Roos *et al*. [114] documented that CLA isomers differentially affect plasma lipid levels as well as the markers of insulin resistance and inflammation in apolipoprotein E knockout mice. The *cis*-9, *trans*-11 CLA isomer lowered these values suggesting beneficial properties, whereas the *trans*-10, *cis*-12 CLA isomer increased the values indicating detrimental properties. In Nestel *et al*'s study [115] using insulin deficient apoE deficient mouse models, 0.9% (final dietary concentration) *cis*-9, *trans*-11 CLA isomer diet failed to reduce the severity of aortic atherosclerosis, though plasma triglyceride levels decreased, and HDL cholesterol levels increased.

#### Hamsters

Like mouse models, hamster models have shown protective effects, some of which are isomer-specific. Hamsters fed a CLA isomer mixture diet (*cis*-9, *trans*-11 CLA isomer: *trans*-10, *cis*-12 CLA isomer = 1:1, final dietary concentrations 0.06, 0.11, and 1.1%) showed significantly reduced plasma levels in total cholesterol, non-high-density lipoprotein cholesterol, and triglycerides [116]. In Wilson *et al*'s study [117] using hamster models, animals on the hypercholesterolemic diet (HCD) supplemented 1% (final dietary concentration) CLA isomer mixture diet (*cis*-9, *trans*-11 CLA isomer: *trans*-10, *cis*-12 CLA isomer = 1:1) showed 47% fewer aortic fatty streaks and lower plasma cholesterol levels than control. In addition, the CLA isomer mixture diet reduced the development of early aortic atherosclerosis to a greater degree than linoleic acid, possibly through changes in LDL oxidation susceptibility in hypercholesterolemic hamsters. Both the *cis*-9, *trans*-11 and *trans*-10, *cis*-12 CLA isomer (1% diet of each isomer) fed groups of hamsters had non-significantly decreased fatty streak lesions. However, neither diet affected plasma cholesterol levels [118]. Wilson *et al*. [119] later reported the adverse effects of the *trans*-10, *cis*-12 CLA isomer, but not the *cis*-9, *trans*-11 CLA isomer, suggesting an isomer-dependent effect of CLA on atherogenesis in hypercholesterolemic hamster models. In the Wilson *et al*'s study, hamsters were divided into four groups and were fed for up to 12 weeks: 1) an HCD, 2) an HCD with 0.5% (of diet) *cis*-9, *trans*-11 CLA isomer, 3) an HCD with 0.5% (of diet) *trans*-10, *cis-*12 CLA isomer, or 4) an HCD with linoleic acid (LA). Both CLA fed groups had lower blood cholesterol levels. However, the *trans*-10, *cis*-12 CLA isomer fed group had higher plasma triglyceride and glucose levels compared with the control at 12-weeks of treatment, while the plasma triglyceride and glucose levels of the *cis*-9, *trans*-11 CLA isomer fed group were reduced. Wilson et al. concluded that the *trans*-10, *cis*-12 CLA isomer may be detrimental if fed separately from the cis-9, trans-11 CLA isomer. In contrast, Navarro *et al*. demonstrated favorable effects of the *trans*-10, *cis*-12 CLA isomer on lipid metabolism in the blood and the liver of hamsters fed an atherogenic diet for 6 weeks and no effects of the *cis*-9, *trans*-11 CLA isomer on the same lipid metabolisms [120]. Studies by Valeille *et al*. [121,122] exhibited the anti-atherogenic and anti-inflammatory effects of the *cis*-9, *trans*-11 CLA isomer in hyperlipidemic hamsters.

#### Inter-/intra-species, tissue-, isomer-specificities

At dietary levels of 0.1–1%, the CLA isomer mixture caused substantial regression of established atherosclerosis in earlier rabbit models [46,47,111]. This was a unique and important finding, because once established, aortic lesions in rabbits will regress only under unusual circumstances. Regression of pre-established lesions has never been achieved by dietary means or by simple pharmacologic intervention *in vivo*. However, the use of rabbit models for atherosclerotic studies may not be suitable. Unlike humans, the majority of rabbit blood cholesterol is β-VLDL. Therefore, the aortic lesions caused by feeding atherogenic diets to rabbits may not be comparable to those seen in humans. In contrast, LDLR- or apo-E-deficient mouse models mimic human atherosclerosis [123]. Thus, the effects of dietary CLA isomer supplementation have not been consistent between these different animal models. Even between rats and mice, a different species response to CLA isomers has been indicated. Any response of peroxisome proliferattion to CLA isomers may be greater in mice than in rats [124]. CLA isomers are known to be PPAR activators [6]. Differences in CLA-mediated hepatic gene induction between mice and rats have also been found [125]. Hepatic fat accumulation caused by the *trans*-10, *cis-*12 CLA isomer has been reported mainly in mice, and hepatic fat accumulation is associated with the loss of adipose tissue induced by the *trans-*10, *cis*-12 CLA isomer [126]. Adipose tissues are important endocrine organs that produce inflammatory mediators such as TNFα and IL-6 and -8, and adipocytokines (adiponectin and leptin). Adipocytokines are key regulators of insulin resistance. Adiponectin and leptin affect immune and inflammatory functions [127]. The dramatic decrease in adiponectin concentrations is important to the development of hepatic steatosis and insulin resistance induced by CLA. Removing CLA from the diet rescued leptin and adiponectin levels and attenuated insulin resistance induced by dietary CLA in mice. However, if a PPARγ activator, rosiglitazone, was added to CLA-TG diet (38.5% *trans*-10, *cis*-12 CLA isomer), the reduction in adipose mass and serum leptin and adiponectin levels was reversed [128].

An opposite response to that found in mice, CLA mixture diet (39.2% the *cis*-9, *trans*-11 and 38.5% *trans*-10, *cis*-12 CLA isomers) reduces hepatic steatosis and plasma lipids in rats [129,130]. Beyond rodent models, the genetic differences between mice and humans should be considered. For example, mice contain more copies of cytochrome P450 than do humans [131]. Cytochrome P450 is involved in microsomal ω-oxidation of fatty acids, eicosanoid synthesis, and detoxification of xenobiotics. The *trans*-10, *cis*-12 CLA isomer significantly reduces cytochrome P450 gene expression in mouse livers, and the reduction may contribute to CLA-induced fatty livers as well as the induction of enzymes associated with fatty acid synthesis [126]. Thus, not only are there differences in CLA-mediated cytochrome P450 gene expression, there are additional differences in fatty acid metabolism and eicosanoid formation, between humans and mice. As described in Section 6, single nucleotide polymorphisms (SNPs) among human individuals, such as SNPs in *APOA1*, *PPARA*, *PPARG*, *SOD2*, *Gpx1*, and *TLR4*, may also cause differences in lipid metabolism and the risk for atherosclerosis. In addition, the age of animals fed CLA and examined may be another consideration. Many atherosclerotic studies used adolescent individuals for their animal models. Adolescent animals are still growing; their body composition is still changing. The gene expression profile and sensitivity to and metabolism of chemicals in a developmental stage differ from those in adults. Such differences may make the extrapolation to humans from animals and explanations of study results more difficult. Thus, there are several factors to be considered when investigating the effects of CLA isomers as therapeutic or chemopreventive agents for atherosclerosis: dose-dependency and isomer-specificity, as well as inter- and intra-species differences. Therefore, genetic and genomic research using human subjects and/or human cells are urgently needed to determine the effects of each isomer.

### Human Studies

CLA is being sold as a panacea with several alleged benefits including altering body composition, *i.e*., to reducing obesity and building lean body mass [132]. Safety of long-term (≥ 12 months) CLA supplementation was examined in several clinical trials. A randomized, double-blind study was conducted, in which obese individuals were given 6 g/day of either CLA isomer mixture (*cis*-9, *trans*-11: *trans*-10, *cis*-12 = 50:50) or placebo (high oleic sunflower oil) for 12 months. Although body composition did not differ between the CLA-supplemented group (n = 27) or the placebo group (n = 23), lower levels of adverse effects (alterations in the liver function, glucose and insulin levels, insulin resistance, and white blood cell counts) were observed in the CLA-supplemented group than in the control group. The investigators concluded that CLA isomer mixture as Clarinol™ is safe for use in obese humans for up to one year at the recommended dosage [133]. Another long-term (one year) CLA isomer mixture supplementation study was performed in a double-blind fashion [134]. Healthy overweight humans (n = 180) were randomly divided into three groups: 1) CLA free fatty acid (*cis*-9,*trans*-11: *trans*-10, *cis*-12 = 50:50; 3.6 g CLA isomers/day as FFA forms), 2) CLA-triacylglycerol group (*cis*-9, *trans*-11: *trans*-10, *cis*-12 = 50:50; 3.4 g CLA isomers/day as TAG forms), and 3) placebo (olive oil). The CLA isomer mixture supplementation decreased body fat mass in healthy overweight adult humans. However, there were significant increases in: LDL levels in the CLA-FFA group, HDL levels in the CLA-TAG group, and lipoprotein levels in both CLA groups. Adverse effects, mostly gastrointestinal, were reported by 11.4% of the subjects, and likely resulted from the daily ingestion of oil or of the gelatin capsule alone. Overall, the adverse effects did not differ significantly between the CLA groups and the placebo group, indicating that CLA isomer mixture was tolerated as well as olive oil as the control. One hundred twenty five of 180 subjects who finished this study, continuously participated in the CLA isomer mixture supplementation study for an additional year, thus, total 2 years [135]. Two-year-CLA isomer mixture supplementation groups significantly reduced body weight, BMI, body fat mass, energy intake and serum leptin levels, compared with the baselines at month 0. However, serum lipoprotein and aspartate amino transferase levels, and whole blood leukocyte and thrombocyte counts were significantly increased in the CLA groups. Gaullier et al. concluded that CLA isomer mixture supplementation for 24 months in healthy, overweight adults was well-tolerated, and that CLA isomer mixture may be beneficial as a weight loss supplement. Another one-year CLA isomer mixture supplementation study [136] was conducted in a randomized, double-blind, placebo-controlled fashion. No significant differences in body weight or body fat regain were observed between the CLA group (*cis*-9, *trans*-11: *trans*-10, *cis*-12 = 50:50; 3.4 g/day as TAG forms; n = 40) and placebo (4.5 g olive oil; n = 43). No significant differences in adverse effects or indexes of insulin resistance were observed between the groups. However, a significant increase in the number of leukocytes was observed in the CLA group. Although the investigators did not obtain a perfect group match for body weight at randomization, they concluded that the CLA isomer mixture supplementation for one year has no preventative effect on body weight and body fat regain after the weight loss induced by a low calorie diet for eight weeks in obese subjects.

Many studies have investigated the effects of short-term (mostly 12 weeks or 8 weeks) CLA supplementation. In a six-month double-blind CLA isomer mixture supplementation study [137], 118 healthy overweight and obese adult humans were randomized into two groups supplemented with either 3.4 g/day CLA isomer mixture (*cis*-9, *trans*-11: *trans*-10, *cis*-12 = 50:50) or placebo. CLA significantly decreased body fat mass, in particular in legs of both males and females and in females with BMI >30 kg/m^2^, at either month 3 or 6, compared with placebo. Lean body mass increased in the CLA supplemented group. The safety parameters including blood lipids, inflammatory and diabetogenic markers remained within the normal range, and adverse events did not differ between the groups in the study. It was concluded that the CLA isomer mixture supplementation in healthy, overweight, and obese subjects decreases body fat mass in specific regions and was well tolerated. The dose-dependent effects of CLA were reported in a CLA isomer mixture supplementation study of 12 weeks [138]. Forty eight obese subjects were divided into three groups: 1) 3.2 g/day CLA (*cis*-9, *trans*-11: *trans*-10, *cis*-12 = 50:50), 2) 6.4 g/day CLA, and 3) placebo (8 g safflower oil). CLA isomer mixture supplementation at the higher dose increased inflammatory markers, IL-6 and C-reactive protein (CRP), however, remained within normal ranges. A significant increase in lean body mass was also found in the same treatment group. No severe adverse effects were reported. The authors concluded that the CLA isomer mixture intervention was well-tolerated. Beneficial effects on immune functions have been reported in a double-blind, randomized CLA isomer mixture supplementation study [139]. Twenty-eight healthy adults received either high oleic sunflower oil (placebo) or 3.0 g/day CLA (*cis*-9, *trans*-11: *trans*-10, *cis*-12 = 50:50; triglyceride form) for 12 weeks. The CLA group showed significantly reduced levels of the proinflammatory cytokines, TNF-α and IL-β, and increased levels of the anti-inflammatory cytokine, IL-10. Immunoglobulin levels were also altered: CLA isomer mixture decreased Ig E levels, and increased both Ig M and Ig A levels. Another CLA isomer mixture supplementation study (2.2 g/day; *cis*-9, *trans*-11: *trans*-10, *cis*-12 = 50:50; 8 weeks) investigated the effects of CLA isomer mixture on inflammation in a double-blind, randomized, placebo-controlled model using healthy middle-aged males [140]. The CLA isomer mixture supplementation significantly reduced concanavalin A-stimulated peripheral blood mononuclear cell IL-2 secretion, suggesting anti-inflammatory and anti-atherogenic effects of CLA isomer mixture. Other inflammatory markers, IL-6, CRP, and fibrinogen, were not affected in this study. Moloney *et al*. [141] demonstrated that the CLA isomer mixture supplementation (3.0 g/day; *cis-*9, *trans*-11: *trans-*10, *cis-*12 = 50:50; 8 weeks) increased total HDL cholesterol concentrations and decreased the ratio of LDL cholesterol to HDL cholesterol without changes in inflammatory markers of CVD in subjects with type 2 diabetes. However, this CLA isomer mixture intervention did not show positive effects on insulin and glucose concentrations among the diabetic patients. In a Swedish study, 53 healthy humans were randomly assigned to CLA isomer mixture supplementation (4.2 g/day; *cis-*9, *trans*-11: *trans-*10, *cis-*12 = 50:50; 12 weeks) in a double-blind fashion. Supplementation with a CLA isomer mixture reduced the proportion of body fat and affected fatty acid metabolism. However, no effects were found for CLA isomer mixture on body weight, serum lipids, glucose metabolism or plasminogen activator inhibitor 1 [142]. In Noone *et al*.'s double-blind placebo-controlled study [143], the CLA isomer mixture treatment (3 g/day; cis-9, trans-11:trans-10, cis-12-CLA = 50:50; 8 weeks) showed reduced plasma triacylglycerol levels in normolipaemic human subjects.

A study using 49 healthy male subjects showed the isomer-/dose-dependent (0.59, 1.19, 2.38 g/day of the *cis*-9, *trans*-11 CLA isomer; 0.63, 1.26, 2.52 g/day of the *trans*-10, *cis*-12 CLA isomer; 8 weeks) opposite effects of CLA on plasma total cholesterol and LDL-cholesterol levels: hypolipidemic properties of the *cis*-9, *trans*-11 CLA isomer and hyperlipidemic properties of the *trans*-10, *cis*-12 CLA isomer. However, neither CLA isomer supplementation affected insulin resistance [144]. Using the same healthy male subjects and the same supplementation design, Tricon *et al*. investigated the effects of two CLA isomers on immune cell functions [145]. The results showed a dose-dependent reduction in the mitogen-induced activation of T lymphocytes and a negative relationship between the mitogen-induced T lymphocyte activation and the contents of each CLA isomer in mononuclear cells, suggesting beneficial effects in inflammatory diseases such as atherosclerosis.

Additional adverse effects of CLA supplementation, in particular *trans*-10, *cis*-12 CLA, were reported. Riserus *et al*. demonstrated that the purified trans-10, cis-12 CLA isomer supplementation (3.4 g/day, 3 months), but not CLA mixture supplementation (3.4 g/day; *cis*-9, *trans*-11: *trans*-10, *cis*-12 = 50:50; FFA form, 3 months), increased oxidative stress, CRP, and proinsulin levels, and decreased insulin sensitivity in non-diabetic abdominally obese males [146] and in males with metabolic syndrome [147] in two double-blind, randomized, placebo- (3.4 g/day, olive oil) controlled studies. Like the study by Tricon et al. [144], Riserus *et al*.'s two studies also suggest the isomer-dependent detrimental effects of CLA. Another unfavorable effect of the *trans*-10, *cis*-12 CLA isomer was also reported in a human study examining non-enzymatic and enzymatic lipid peroxidation (8-iso-PGF_2α _and 15-keto-dihydro-PGF_2α, _respectively) in human plasma and urine. Sixty healthy subjects were divided into six groups: three CLA isomer mixture groups, (3.5 g/day, *cis*-9, *trans*-11: *trans*-10, *cis*-12 = 50:50, 4 weeks) and three trans-10, cis-12 CLA isomer groups (4.0 g of the *trans*-10, *cis*-12 CLA isomer/day, 4 weeks): 1) the CLA supplement alone, 2) with vitamin E (D-α-tocopherol acetate), and 3) with COX-2 inhibitor (refecoxib). Although both CLA isomer mixture and the *trans*-10, *cis*-12 CLA isomer supplementations increased the eicosanoid levels in the urine, the *trans*-10, *cis*-12 CLA isomer supplementation with the COX-2 inhibitor suppressed the increase in urinary 15-keto-dihydro-PGF_2α _levels. This result suggests that increased lipid peroxidation in eicosanoid synthesis may be due to induced COX-2 expression by the CLA supplementations, in particular the *trans*-10, *cis*-12 CLA isomer [148]. Taylor *et al*. [149] documented that CLA isomer mixture supplementation (4.5 g/day; *cis*-9, *trans*-11: *trans*-10, *cis*-12 = 50:50; 12 weeks) impaired endothelial function and increased markers of oxidative stress in 40 healthy white males, suggesting caution in the use of CLA isomers as an aid for weight loss.

Overall, the effects of CLA isomers (or mixture) on atherogenic and/or inflammatory parameters in humans have not been definitive. Although a meta-analysis of 18 CLA human studies (including three single isomer studies) suggests the beneficial use of CLA isomers only as a body fat reducing supplement [150], the therapeutic potentials of CLA isomers in inflammatory diseases including atherosclerosis remain to be determined. Differences in purity and content of CLA isomers may cause these conflicting results. Impurities might induce undesirable side effects [151]. The variety of CLA isomer content in supplements and/or the differences in CLA dose might cause inconsistent results due to the dose- and/or isomer-dependent effects of CLA suggested by some other studies [138,144,146,147]. Human subjects were not limited in diet (therefore, dietary fat intake, excluding supplemental fat intake, is of concern) and/or physical activities in some study designs. In addition, CLA isomer (or mixture) supplementations contained other fatty acids, including PUFAs and saturated fatty acids, at up to 20% in some study designs. The details of supplemental contents, other than CLA isomers, are not even provided in some other studies. Since CLA isomers are incorporated into membrane phospholipids, they may compete in enlongation and desaturation steps with other PUFAs that are precursors of arachidonic acid (AA, 20:3, ω-6). The competition in the incorporation may alter eicosanoid biosynthesis [152], therefore, subsequent immune and inflammatory processes. An experiment by Brown *et al*. [153] found that the presence of linoleic acid (LA) may affect the possible benefits of CLA isomers. LA, one of the PUFAs present in Western diets and the human body is a possible antagonist to CLA isomers. The plentiful LA may exclude CLA isomers from incorporation into phospholipids and drive it into storage as a component of neutral lipid [52]. LA also inhibits EPA incorporation in membrane phospholipids from fish-oil supplements [54]. Like CLA isomers, dietary EPA and DHA partially replace AA derived from LA in the cell membrane. These ω-3 fatty acids are associated with decreased risk for CVD, and reduce the formation of pro-inflammatory eicosanoids [53]. The replacement of AA by ω-3 fatty acids may cause the alteration of fatty acid composition in membrane lipid bilayers influencing signaling pathways and subsequent immune and inflammatory processes. The anti-inflammatory effects of CLA isomers observed in a limited number of studies may be attributed to the replacement of AA generated by LA. In addition, saturated fatty acids have been reported to provoke inflammation by inducing pro-inflammatory gene expression through innate immune receptor (TLRs) activation [61]. Thus, the coexistence of other fatty acids might potentially affect the results of any human studies with CLA isomers. Moreover, there may be divergent effects of CLA isomers in obese or diabetic subjects compared to the normal-weight or healthy subjects as well as differences determined by gender and/or genetics, *i.e*., SNPs in related genes. Further studies are needed to investigate the effectiveness and safety of CLA supplementation and to elucidate these confounding factors.

### CLA mediated gene expression

*Adiposity plays an important role in fatty *acid mobilization, fat storage, and formation of pro-and anti-inflammatory cytokines and chemokines [128]. As mentioned above, atherosclerosis is viewed as a chronic inflammatory disease affecting lipid profiles. CLA isomers have been shown to influence lipid metabolism associated with inflammation and atherogenesis in *in vitro *studies. One possible mechanism by which CLA isomers could modulate atherogenesis is regulating the production of lipoproteins in the liver. Sterol element binding proteins (SREBP) are a group of membrane-bound transcription factors that bind to their specific DNA binding sites (SRE-1) to activate the expression of target genes that encode enzymes necessary for lipid synthesis, including the LDL receptor (LDLR) gene in sterol-depleted cells [154]. A study by Ringseis et al. [155] reported that the *trans-*10, *cis*-12 CLA isomer, not the *cis*-9, *trans*-11 CLA isomer, induced LDLR gene expression via SREBP-2 in human hepatoma cells (HepG2). They concluded that the enhanced uptake of VLDL and LDL cholesterols by hepatic LDLR may account for the decreased plasma cholesterol levels in response to CLA isomer (or mixture) supplementations in a limited number of human and animal studies. An alternative pathway for CLA-mediated LDLR expression is also suggested. Yu-Poth et al. [156] demonstrated that a CLA isomer mixture (50:50, 400 μmol/L final concentration) up-regulated LDL receptor (LDLR) mRNA and protein expression at three- to five-fold in HepG2 cells. The results of the study suggest the upregulation of the LDLR gene by CLA through a mechanism that is independent of SREBP-1 and acyl CoA: cholesterol acyltransferase (ACAT).

Monocyte-endothalial interaction is a key step of atherogenesis. However, CLA isomers showed no effects on TNFα-induced adhesion molecule expression, monocyte adhesion, and chemokine release or on the molecular mechanisms regulating these processes in human aortic endothelial cells [21]. This suggests that the anti-atherogenic effects of CLA isomers may not be associated with the reduction of monocyte-endothelial interactions.

Several studies have investigated the implication of CLA isomers in eicosanoid synthesis and the role of CLA isomers in inflammation and atherogenesis. Dietary CLA may suppress the biosynthetic pathway of AA. CLA may suppress eicosanoid formation via direct action on COX and LOX, *i.e*., by inhibiting the expression or the activities of these enzymes [5,6]. Constitutive COX-1 and inducible COX-2 catalyze the conversion of free PUFAs to prostanoids, while LOX generates the leukotrienes. The *trans*-10, *cis*-12 CLA isomer suppresses COX-2 expression and PGE_2 _release in rat macrophages either by inhibiting NF-κB activation *in vivo *and *in vitro *or by inhibiting the MAPK/ERK/JNK pathway. The trans-10, cis-12 CLA isomer inhibits the NF-κB p50 and p65 subunits binding to DNA [83]. The 50:50 mixture of CLA isomers inhibits the expression of both COX-2 and inducible nitric oxide synthase (iNOS) in LPS activated murine macrophages, resulting in decreases in prostaglandin E_2 _and NO synthesis [157]. The effects of CLA on prostanoid formation can be either inhibitory or stimulatory, depending on isomer-specificity, chemical forms of CLA isomers (*i.e*., free fatty acid or esterified forms), or cellular states (i.e., resting or stimulated states) in human endothelial cells and platelets [158]. CLA isomers have also been reported to reduce prostaglandin E2 synthesis in certain cell types in both humans and mice [159].

The beneficial effects of CLA isomers may be attributed to their properties as PPARα/γ activators [5,48,155,160]. Structurally, CLA resembles 13-HODE, as well as 15-HETE and 15d-PGJ2, which were all identified as natural activators of PPARγ [49]. Ringseis et al[160] demonstrated that either the *cis*-9, *trans*-11 or *trans*-10, *cis*-12 CLA isomer (50 μmol/L) reduced AA proportions in human vascular smooth muscle cells (SMCs), TNFα-induced NF-κB DNA binding activity, mRNA levels of enzymes involved in eicosanoid synthesis (*e.g*., COX-2), and production of PGE2 and PGI2. These CLA isomer treatments increased PPARγ DNA binding activity. Furthermore, a PPARγ repressor suppressed the inhibitory actions on the eicosanoid formation and NF-κB DNA binding activity in the SMCs. Synthetic PPAR activators exert their anti-inflammtory actions, at least in part, by negatively regulating NF-κB activation [157]. PPARγ and NF-κB may be involved in regulating genes, whose products influence ROS generation that contributes to inflammation and atherogenesis, and these transcription factors coordinate and propagate feedback loops between each other. Thus, anti-inflammatory and anti-atherogenic effects of CLA isomers may be associated with these redox-sensitive transcription factors.

Although CLA isomers and ω-3 PUFAs have shown anti-inflammatory effects, they differ in the nature of their immunomodulatory properties. CLA isomers appear to enhance immune function, while ω-3 PUFAs are immunosuppressive [5]. Tian *et al*.'s study [51] using rats treated with 300 mg/kg/day of clofibrate, a PPARα activator, for up to 14 days, reported an increase in myocardial DHA proportion and a decrease in the portion of AA that is a precursor of pro-inflammatory eicosanoids (*e.g*., leukotriene B4 and prostaglandin E2). Tian *et al*. implicate enhanced uptake of ω-3 PUFAs from blood circulation and/or increased biosynthesis of ω-3 PUFAs in rats treated with the PPARα activator, in those results. Similarly, Attar-Bashi *et al*.'s human study [161] investigated the effects of CLA isomers mixture [3.2 g/day, *cis*-9, *trans*-11: *trans*-10, *cis*-12 = 50:50, plus 11 g of alpha-linoleic acid (ALA), 8 weeks] as PPARα activators on DHA (22:6, ω-3) and EPA (20:5, ω-3) biosynthesis from ALA (18:3, ω-3) through Δ5- and Δ6-desaturases, both of which are possible PPARα target genes. The study demonstrated that ALA (18:3, ω-3) plus CLA isomer mixture increased EPA and decreased AA. However, the CLA isomer mixture supplementation did not affect DHA biosynthesis in humans. DHA synthesis from ALA needs additional peroxisomal oxidation. Thus, CLA isomers may play a role in PPARα-mediated gene expression and ω-3 PUFA-mediated anti-inflammatory effects.

CLA isomers are readily metabolized *in vivo *via multiple pathways, and enlongated and desaturated metabolites of CLA have been detected in the liver and mammary tissue of rats and adipose tissue and sera of humans [6]. Some studies have suggested the involvement of CLA metabolites in anti-atherogenic and ant-inflammatory processes [5,160], though the Δ6-desaturase metabolites of CLA may not be important for the alterations in gene expression induced by CLA [6].

Thus, multiple signaling pathways, such as PPARα, PPARγ, NF-κB, and MAPK/ERK/JNK, may be involved in the anti-inflammatory and anti-atherosclerotic effects of CLA isomers.

## Conclusion

Conjugated linoleic acid isomers are a group of zoochemicals that have a variety of physiological actions and potential health benefits, *e.g*., modulation of inflammation, lean body mass, atherosclerosis, and cancer. Most of the health effects of CLA isomers are based on reports with limited power of extrapolation of experiments with animal models and *in vitro*/*ex viv*o systems. Multiple factors in earlier animal studies might cause inaccurate extrapolation of CLA isomer effects to humans. First, interspecies-genetic differences may be a major consideration. Some of the health effects of CLA isomers seen in animal models might be species-specific, and might not be observable in humans. For example, the suppressive effect on hypertension was seen in CLA fed rats [162-165], but not in humans [166]. Second, animal studies used primarily adolescent animals, while human studies used mostly healthy, obese, or diabetic adults. Body composition, sensitivity to and metabolism of chemicals, and gene expression profiles in developing adolescent animals may be different from those seen in adult humans. This could influence the outcome of examining the effects of CLA isomers, in particular, on age-associated events, such as adiposity and atherogenesis. Although atherosclerosis-prone mice (*i.e*., C57BL/6) mimic humans, animal results have been inconclusive and the efficacy of CLA isomers maybe due to interspecies-gene differences. Third, animal studies have usually been of short durations with high dosages. This is of particular concern because extrapolated CLA isomer dosages for possible human consumption would require dose and duration adjustments for human lifespan and levels at or less than threshold toxicity.

The effects of CLA isomers on body composition have been studied extensively in animals and have recently been repeated in human studies with conflicting findings. In part, the results may be inconclusive because a majority of the studies utilized CLA isomer mixture supplementation. Both dose- and isomer-dependent effects of CLA have been suggested in both the animal and human studies. Further research is needed to identify isomer-and/or dose-related efficacy and toxicity. In addition, recent studies have identified SNPs in genes related to lipid metabolism and antioxidant defense systems. It may be necessary to investigate the effects of CLA isomers on CVD risk at intra-species levels. Some recent animal studies have reported a positive correlation with other disease prevention and treatment, *e.g*., diabetes and inflammatory bowel disease [48,49,128,129] leading researchers to further evaluate the possible benefits of CLA isomer supplementation for humans.

Earlier research showed that the ability of CLA isomers to act as anti-carcinogens and protectants against atherosclerosis may be due to its role as an antioxidant. However, in light of current research, the modulation of chronic diseases by CLA isomers may involve the control of redox status by regulating genes, whose products influence ROS generation, through redox-sensitive transcription factors including PPARγ and NF-κB. It is essential that investigation to develop an understanding about the molecular action of CLA isomers be encouraged so that we may learn how to use these compounds as adjuvants in chronic disease therapy.

## Competing interests

The authors declare that they have no competing interests.

## Authors' contributions

YKN, NF-D and STO designed and wrote this manuscript. YKN and NF-D conceived and participated in the design and coordination of the literature review. All authors read and approved the final manuscripts.
